# Exercise-induced modulation of astrocyte in Alzheimer’s disease: mechanisms and therapeutic implications

**DOI:** 10.3389/fphys.2026.1827919

**Published:** 2026-05-07

**Authors:** Lei Shi, Jiding Xie, Tianjiao Dai, Mingzheng Zhang, Taoshuo Yang, Limei Sheng, Qiguan Jin, Jingang Dai

**Affiliations:** 1 College of Physical Education, Yangzhou University, Yangzhou, Jiangsu, China; 2 Experimental Research Center, China Academy of Chinese Medical Sciences, Beijing, China; 3 School of Rehabilitation, Jiangsu College of Nursing, Huai’an, China; 4 College of Medical, Veterinary and Life Sciences, University of Glasgow, Glasgow, United Kingdom

**Keywords:** A1/A2 polarization, Alzheimer’s disease, AQP4, astrocytes, exercise intervention, neuroinflammation

## Abstract

Alzheimer’s disease (AD) is characterized by extracellular amyloid-β (Aβ) deposition, tau pathology, and chronic neuroinflammatory responses, although the relative contribution of these processes varies across disease stage and patient population. Current pharmacological therapies provide limited symptomatic benefit or modest disease-slowing effects in selected populations, underscoring the need for safe and sustainable adjunctive interventions. Astrocytes are central regulators of synaptic homeostasis, metabolic support, vascular coupling, and perivascular solute clearance, and these functions are profoundly altered in AD. For heuristic purposes, reactive astrocytes are often described along an A1-like to A2-like spectrum, with the former associated with pro-inflammatory and neurotoxic programs and the latter with reparative and neurotrophic functions; however, emerging single-cell and spatial transcriptomic data indicate that astrocyte states in AD are heterogeneous and context dependent rather than strictly binary. Growing evidence, predominantly from preclinical studies, suggests that exercise may remodel astrocyte-associated inflammatory, metabolic, and clearance pathways, with potential benefits for AD-related pathology and cognition. In several rodent models, exercise has been associated with reduced expression of A1-like reactive markers, enhanced protective astrocyte-associated programs, improved astrocyte–neuron metabolic coupling, and better perivascular localization of aquaporin-4 (AQP4). These changes may contribute to reduced inflammatory signaling and more efficient clearance of Aβ and tau, although the extent to which AQP4-dependent perivascular clearance mechanisms mediate exercise benefits in humans remains uncertain. Here, we review current evidence on how exercise influences astrocyte biology in AD, distinguish preclinical from clinical findings, and discuss key translational modifiers—including exercise modality, disease stage, sex, and APOE genotype—to inform glia-aware therapeutic strategies and future exercise prescriptions.

## 1 Introduction

Alzheimer’s disease (AD) is clinically characterized by progressive cognitive decline and memory impairment, but its pathogenesis is multifactorial and not fully explained by any single pathogenic cascade (Baviskar and Mahajan, 2025). Neuropathologically, the canonical hallmarks of AD include extracellular senile plaques formed by excessive deposition of amyloid-β (Aβ) and neurofibrillary tangles composed of abnormally phosphorylated tau. These protein aggregates are widely believed to drive neuronal loss and synaptic degeneration, thereby precipitating cognitive deterioration (Taj et al., 2025). In particular, soluble Aβ oligomers have been shown to exert strong neurotoxicity, disrupting synaptic glutamatergic transmission and impairing synaptic function (Bai et al., 2025). Beyond proteopathic aggregation, pathological Aβ and tau can activate innate immune responses in the brain and induce chronic neuroinflammation (Butovsky et al., 2025). Neuroinflammation is now recognized as a major feature of AD, with activated microglia and astrocytes releasing pro-inflammatory mediators that accelerate neurodegenerative changes (Shi et al., 2025; Wang et al., 2025; Zhou and Glass, 2025). Unfortunately, owing to the heterogeneity and complexity of AD pathogenesis, effective curative treatments remain elusive. For example, several candidate agents, including AL002 and simufilam, have not yet demonstrated consistent clinical benefit, and even recently developed anti-Aβ therapies provide only modest slowing of decline in selected patients (Piller, 2024). After repeated failures in drug development, even the latest anti-Aβ antibody therapies provide only modest slowing of disease progression and fail to halt neurodegeneration. Consequently, there is a pressing need to explore alternative or complementary intervention strategies.

In recent years, astrocytes have emerged as major mediators of AD-related inflammation, metabolic dysregulation, and clearance failure, making them attractive—yet mechanistically complex—therapeutic targets (Serrano-Pozo et al., 2024; La Barbera et al., 2025; Rabah et al., 2025). Astrocytes, the most abundant glial cells in the central nervous system, perform diverse supportive and protective functions under physiological conditions (Carter et al., 2019). Through highly ramified processes, astrocytes contact tens of thousands of synapses and blood vessels, maintaining extracellular homeostasis, regulating neurotransmitter balance, and providing metabolic substrates (Zhang et al., 2025). Astrocytes supply lactate and other metabolites to active neurons and are integral to the “neuron–glia–vascular” functional unit, coupling neuronal activity with metabolic support. Astrocytes are also crucial for waste clearance: aquaporin-4 (AQP4) channels at astrocytic endfeet drive glymphatic clearance of Aβ and pathological tau from the brain parenchyma (Feng et al., 2023; Li et al., 2025). Accordingly, astrocytes are indispensable for maintaining brain homeostasis and preventing toxic waste accumulation. However, in the AD brain, astrocytes undergo pronounced reactive changes, with their morphology and functional phenotypes dramatically altered. In AD patients and models, astrocyte numbers may not dramatically increase, yet these cells display hypertrophy—manifested by thickened processes and elevated glial fibrillary acidic protein (GFAP) levels—along with a reorientation of their processes toward amyloid plaques (Smith et al., 2019; Wang et al., 2022). These aberrant astrocytic responses are thought to contribute to multiple pathological processes — including chronic neuroinflammation, synaptic dysfunction, and disrupted brain energy metabolism — thereby accelerating neurodegeneration.

Astrocytes can exert both detrimental and protective effects in AD, but these responses are better understood as context-dependent reactive states rather than a simple binary switch (Von Bernhardi and Ramirez, 2001). On one hand, reactive astrocytes release large quantities of pro-inflammatory cytokines, complement C3, and reactive oxygen species, driving a neurotoxic A1-like phenotype. A1 astrocytes are a reactive subtype with canonical neurotoxic features: they overexpress C3 and other pro-inflammatory genes and can induce apoptosis or functional impairment in neighboring neurons (İlğun and Çakır, 2025; Zhou et al., 2025). Conversely, a subset of astrocytes mounts neuroprotective responses, often classified as A2-like phenotypes. A2 astrocytes secrete neurotrophic factors and anti-inflammatory mediators that help mitigate the harmful consequences of excessive glial activation (King et al., 2020; İlğun and Çakır, 2025). For heuristic purposes, AD astrocyte responses are often discussed along an A1-like to A2-like spectrum; however, recent single-cell and spatial transcriptomic studies indicate that reactive astrocytes occupy a multidimensional continuum with partially overlapping inflammatory, metabolic, and vascular programs (Green et al., 2024; Serrano-Pozo et al., 2024; İlğun and Çakır, 2025). Given the central role of astrocytes in inflammation, metabolism, and clearance, targeting astrocyte phenotypes is increasingly viewed as a promising therapeutic avenue in AD.

Among emerging AD management strategies, lifestyle interventions have proven particularly compelling (Halloway et al., 2025; Mackey-Alfonso and Barrientos, 2025). Epidemiological studies and some clinical trials suggest that sustained physical activity is associated with lower AD risk or slower functional decline, although effect sizes vary and the mediating mechanisms in humans remain incompletely defined (Dong et al., 2025; Nguyen Ho et al., 2025; Wang et al., 2025). Regular exercise benefits cardiovascular and metabolic health, and it has been shown to reduce AD pathological burden — for example, by decreasing Aβ plaque deposition and improving memory performance. Compared to pharmacotherapy, exercise is feasible for long-term adherence and may confer broader physiological benefits. At the neurobiological level, exercise promotes the expression of multiple neurotrophic factors (including brain-derived neurotrophic factor), improves cerebral blood flow and synaptic plasticity, and modulates immune-inflammatory responses (Manser et al., 2025; Wang et al., 2025). Importantly, recent preclinical work suggests that exercise may influence astrocyte phenotypes and functions, thereby altering inflammatory tone, metabolic support, and perivascular clearance pathways. Exercise can shift astrocytes from neurotoxic A1-like states toward neuroprotective A2-like phenotypes, and it enhances perivascular AQP4 polarization at astrocytic endfeet, thereby accelerating glymphatic clearance of Aβ and phosphorylated tau (Feng et al., 2023). Exercise also upregulates astrocytic AQP4 expression and restores its polarized localization, thereby improving glymphatic clearance efficiency (Rodríguez et al., 2013; Belaya et al., 2020). Together, these findings support the hypothesis that exercise can remodel astrocyte-associated pathways—including reactive phenotypes, metabolic coupling, and immune signaling—to create a microenvironment more permissive for neuronal resilience, while direct evidence for such mechanisms in humans remains limited.

Accordingly, this review synthesizes mechanistic evidence linking exercise and astrocyte biology in AD, explicitly distinguishes preclinical from clinical observations, and outlines the major translational gaps that must be addressed before evidence-based exercise prescriptions can be refined.

## 2 Alzheimer’s disease

AD is increasingly conceptualized as a multifactorial disorder in which proteostatic failure, neuroinflammation, synaptic dysfunction, metabolic dysregulation, and vascular impairment interact dynamically rather than unfold as a single linear cascade (Sun et al., 2023; Jia et al., 2024; Preis et al., 2024). The following subsections therefore discuss these processes as interdependent components of disease progression.

### 2.1 Pathogenetic mechanisms of Alzheimer’s disease

#### 2.1.1 Proteostasis disruption and pathological aggregation

One of the most central pathological features of AD is excessive generation and aggregation of Aβ peptides. Aβ is produced from the amyloid precursor protein (APP) via sequential cleavage by β- and γ-secretases (Tachida et al., 2023). Under healthy conditions, Aβ is continuously generated but efficiently cleared; in AD, this dynamic balance is disrupted, leading to excessive accumulation. Soluble Aβ oligomers progressively assemble into fibrils and ultimately form insoluble extracellular plaques. Excessive Aβ accumulation disrupts neuronal communication, induces oxidative stress, and activates innate immune responses, resulting in synaptic dysfunction, neuronal injury, and cognitive decline (Ng et al., 2023). Neurofibrillary tangles formed by abnormal tau represent another major pathological hallmark of AD. Tau is a microtubule-associated protein in neurons; in AD, tau becomes abnormally hyperphosphorylated, dissociates from microtubules, and aggregates into paired helical filaments and tangles (Stein-O'Brien et al., 2025). Loss of tau’s normal function destabilizes the neuronal cytoskeleton, impairs axonal transport, and ultimately causes synaptic and neuronal dysfunction. The density and anatomical distribution of neurofibrillary tangles strongly correlate with the severity of neurodegeneration and cognitive impairment, highlighting tau’s critical role in disease progression (Moscoso et al., 2025). Although traditional views propose that Aβ accumulation triggers tau pathology, some evidence indicates that tau pathology can progress relatively independently and may even precede Aβ in certain contexts (Arnsten et al., 2025). Together, disrupted Aβ and tau proteostasis constitutes a central pathological axis in AD, but its effects are best understood in interaction with inflammatory, synaptic, metabolic, and vascular abnormalities rather than as an isolated upstream trigger.

#### 2.1.2 Neuroinflammation and the impact of glial polarization

Beyond proteostatic abnormalities, AD is also characterized by persistent neuroinflammatory responses involving both microglia and astrocytes, which increasingly appear to contribute to disease progression (Sun et al., 2023; Lee et al., 2024). In early stages, glial activation may be protective, aimed at clearing Aβ and cellular debris. However, with sustained disease progression and chronic stimulation, glia undergo phenotypic polarization toward pro-inflammatory states (Mallach et al., 2024).

Microglia play dual and stage-dependent roles in AD. In early disease, they may help contain pathology by clearing aggregates and cellular debris; with persistent injury signaling, however, they adopt more pro-inflammatory and less phagocytic states, although these responses remain heterogeneous and context dependent (Taj et al., 2025). In this state, microglia release large amounts of pro-inflammatory cytokines (such as TNF-α, IL-1β, IL-6), chemokines, reactive oxygen species, and nitric oxide, establishing a sustained inflammatory milieu (Lin et al., 2022). This chronically activated profile is neurotoxic, damaging neurons and synapses and promoting AD progression. Meanwhile, microglial phagocytic and clearance capacity becomes progressively impaired under chronic activation, reducing the efficiency of clearing Aβ and debris.

Astrocytes also participate deeply in AD-related neuroinflammation. In AD brains, astrocytes adjacent to amyloid plaques exhibit pronounced reactive gliosis, including hypertrophy and upregulation of GFAP and inflammatory mediators (Gomes et al., 2024; McComish et al., 2024). In acute injury, such reactive changes may have transient protective significance; however, in chronic AD contexts, they contribute to sustained pro-inflammatory microenvironments (Bellaver et al., 2023). Reactive astrocytes in AD can produce IL-1β, TNF-α, and a range of cytokines and chemokines, amplifying neuroinflammation and directly injuring neurons and synapses (Rostami et al., 2021). Moreover, their uptake and clearance of extracellular Aβ decreases, exacerbating plaque deposition and associated toxicity.

Crosstalk between microglia and astrocytes further amplifies inflammatory cascades: pro-inflammatory signals released by activated microglia can induce astrocytes to transition into neurotoxic states, whereas astrocyte-derived inflammatory factors can in turn activate and recruit more microglia (Ferrari-Souza et al., 2025). This vicious cycle accelerates synaptic loss and neuronal death, beyond the baseline proteopathic burden of Aβ and tau. Thus, AD pathogenesis reflects a convergence of factors: on top of classical protein pathology, innate immune and inflammatory responses driven by glial dysfunction jointly propel neurodegenerative changes.

#### 2.1.3 Synaptic dysfunction and metabolic dysregulation

Synaptic loss is among the strongest correlates of early cognitive decline in AD and often precedes overt neuronal death (Tzioras et al., 2023). Soluble Aβ oligomers can directly bind postsynaptic receptors, impairing synaptic transmission (Prikhodko et al., 2024). Sustained elevation of inflammatory mediators in AD can further compromise synaptic potentiation/LTP and promote neurotoxic excitatory–inhibitory imbalance, aggravating synaptic dysfunction (Almazán et al., 2025).

AD brains show widespread dysregulation of neurotransmitter systems. Degeneration of cholinergic neurons and reduced acetylcholine levels significantly contribute to memory deficits and constitute a major target of current symptomatic therapies (Cen et al., 2025). In addition, glutamatergic dysregulation and impaired inhibitory neurotransmission may lead to network hyperexcitability; notably, BACE1-dependent cleavage of GABA_𝐴_ receptor β subunits reduces inhibitory currents and promotes neural hyperexcitability and disease progression in AD models and human tissue (Bi et al., 2025).

Concomitant metabolic abnormalities are evident, especially in brain energy metabolism. AD is associated with impaired brain energy metabolism involving reduced glucose uptake/utilization (including altered glucose transporter–related processes), disrupted glycolysis and TCA-cycle–linked ATP generation, and mitochondrial dysfunction with impaired oxidative phosphorylation and increased oxidative stress (Yuan et al., 2024). Lipid metabolism is also closely linked to AD risk and progression. APOE4, a major genetic risk factor for late-onset AD, drives astrocytic lipid droplet abnormalities (fewer/larger droplets enriched in unsaturated triglycerides), impaired lipid droplet turnover, and increased sensitivity to lipid peroxidation, potentially exacerbating lipotoxic stress and glial dysfunction (Windham et al., 2024).

#### 2.1.4 Blood–brain barrier disruption and lymphatic–metabolic clearance dysfunction

Integrity of the blood–brain barrier (BBB) is essential for maintaining CNS microenvironmental homeostasis. In AD, blood–brain barrier (BBB) disruption is an early and persistent pathology (Nehra et al., 2022). Damage to endothelial tight junctions, loss of pericytes, and reduced coverage or polarity of astrocytic endfeet all increase BBB permeability. Consequently, peripheral neurotoxic molecules (e.g., fibrinogen, thrombin) and immune cells can infiltrate the brain, exacerbating inflammation and neurodegeneration (Montagne et al., 2015; Sweeney et al., 2018). Meanwhile, BBB dysfunction also impairs Aβ clearance from the brain to the bloodstream (for instance, via reduced LRP1 transporter function), thereby accelerating amyloid deposition. Some investigators emphasize the concept of a “glial–vascular unit/neurogliovascular unit,” highlighting that the BBB and intracranial lymphatic clearance form an integrated functional system (Cortes-Canteli et al., 2010). BBB dysfunction in AD is closely coupled to impaired perivascular and meningeal lymphatic clearance. Reduced efficiency of these drainage pathways may promote retention of Aβ, tau, and inflammatory mediators, thereby reinforcing vascular dysfunction and neuroinflammation in a self-perpetuating cycle (Cortes-Canteli et al., 2010; Natale et al., 2021).

### 2.2 The role of astrocytes in Alzheimer’s disease

**Figure 1:**
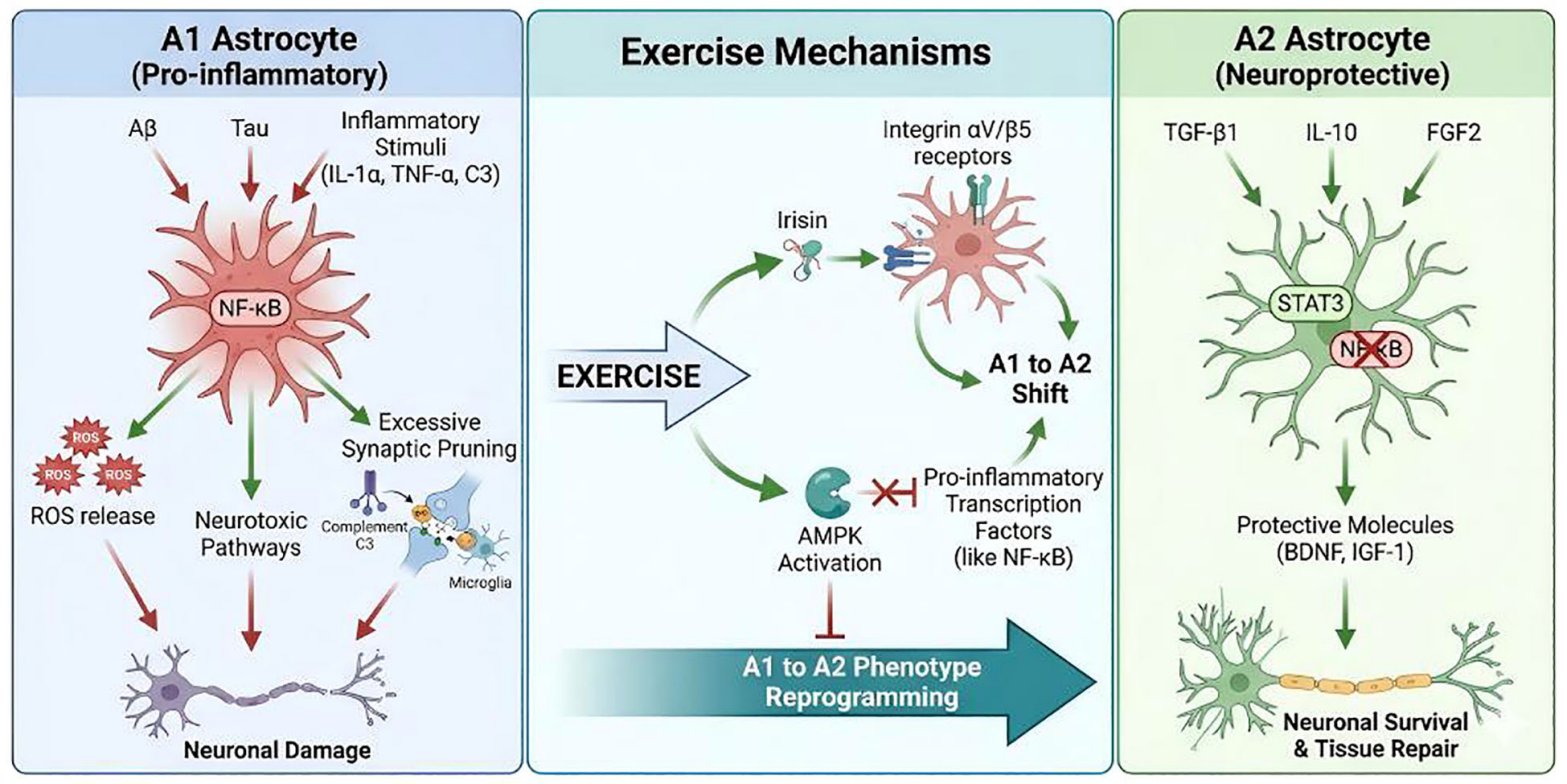
Schematic representation of A1-like and A2-like astrocyte-associated features in Alzheimer’s disease. The A1/A2 framework is used here as a simplified conceptual model; *in vivo* astrocyte states are heterogeneous, overlapping, and context dependent.

Astrocytes are the most abundant and functionally diverse glial cell type in the CNS (Bellaver et al., 2023). They act as “housekeepers” of brain homeostasis, supporting ion balance, metabolic supply, synaptic regulation, and BBB maintenance (Dai et al., 2023). In AD, astrocytes undergo substantial morphological, molecular, and functional remodeling, often referred to as reactive astrogliosis(Reactive Astrogliosis) (Chiotis et al., 2023). This shift is not a uniform pathological event but rather reflects complex phenotypic heterogeneity and functional reprogramming, producing bidirectional and stage-dependent effects on AD progression (Serrano-Pozo et al., 2024) (A brief schematic illustration is provided in **Figure 1**).

#### 2.2.1 Astrocyte polarization states

Under physiological conditions, astrocytes sustain neuronal health by regulating neurotransmitter uptake and recycling, providing trophic support, maintaining ionic and water homeostasis, and promoting neurovascular coupling. Within AD pathology, astrocytes display high plasticity. Acute reactive astrogliosis initially can be neuroprotective, limiting damage and restoring homeostasis (Boghdadi et al., 2020), whereas chronic astrocyte activation in AD can promote persistent neuroinflammation, synaptic dysfunction, and neuronal loss (Kaur et al., 2019). Their reactive state can polarize into distinct functional phenotypes depending on environmental cues, most commonly categorized as neurotoxic A1 and neuroprotective A2. Throughout this review, the A1/A2 terminology is used as a simplified conceptual framework to organize the literature, not as a comprehensive classification of astrocyte states *in vivo*.

##### 2.2.1.1 A1 astrocytes

In AD microenvironments, A1-like astrocyte programs are promoted by sustained proteostatic stress and inflammatory signaling. Protein aggregates act as endogenous danger signals (DAMPs), enhancing microglial and immune-derived mediators such as IL-1α, TNF-α, and complement C1q that can drive astrocytes toward neurotoxic states (Liu et al., 2025). Moreover, inflammatory factors such as lipopolysaccharide (LPS) and IFN-γ, as well as AD-related protein aggregates, can chronically activate astrocytes via pathways including TLR4/NF-κB. Reduced function of antioxidant transcription factor Nrf2 diminishes restraint over NF-κB–mediated pro-inflammatory cascades, further sustaining and expanding A1 polarization (Fan and Huo, 2021; Nakano-Kobayashi et al., 2023). Together, these signals can drive astrocytes from transient adaptive reactivity toward stable, self-amplifying A1 pro-inflammatory phenotypes.

Phenotypically, A1 astrocytes exhibit prominent morphological and molecular changes: enlarged soma and hypertrophic processes, robust upregulation of GFAP, S100B, and complement component C3, and accumulation around amyloid plaques in AD patients and animal models (Kim et al., 2025). Transcriptomically, complement cascade genes and stress/inflammation programs are upregulated, while “homeostatic genes” related to synaptic support and metabolic stability are downregulated (Dai et al., 2023). Functionally, A1 astrocytes release TNF-α, IL-1β, IL-6, NO, ROS, long-chain saturated lipids, D-serine, and other neurotoxic mediators, activate complement-mediated aberrant synaptic pruning, promote excitotoxicity and mitochondrial injury, and ultimately contribute to synaptic loss and neuronal apoptosis (Hinkle et al., 2019). Additionally, impaired expression and function of glutamate transporters in A1 astrocytes reduces clearance of extracellular glutamate and weakens metabolic support (Dang et al., 2024), shifting astrocytes from protectors to amplifiers of neuroinflammation and neurodegeneration.

##### 2.2.1.2 A2 astrocytes

In contrast, A2 astrocytes are generally regarded as anti-inflammatory and neuroprotective, often driven by upregulation of repair-associated signals. In acute ischemia or traumatic injury models, cytokines such as IL-4, IL-10, and TGF-β, along with signaling molecules including TGF-β1/BMP4, FGF2, and PK2, can promote A2 polarization by suppressing NF-κB–mediated pro-inflammatory cascades and activating pathways such as STAT3 and Nrf2, thereby increasing protective genes and A2 markers (e.g., PTX3, SPHK1, TM4SF1) (Neal et al., 2018; Shi et al., 2020). In AD, although chronic inflammation and sustained Aβ load tend to favor A1 polarization, A2-like reactive astrocytes can still be observed, particularly in earlier stages or at lesion margins. Fasudil has been reported to “reprogram” microglia-induced A1 astrocytes into A2 phenotypes, increasing astrocyte-derived neurotrophic factors and producing neuroprotection (Guo et al., 2020). These findings suggest that reactive astrocyte states in AD are dynamically regulated across space and time, and that A1/A2 terminology should be interpreted as an organizing shorthand rather than a strict biological taxonomy.

A2 astrocytes are characterized by preserved phagocytic/metabolic support capacity and increased secretion of neurotrophic and anti-inflammatory factors, buffering AD pathology on multiple levels. Experimental work indicates that A2 astrocytes upregulate genes related to neuronal survival and axonal regeneration (e.g., Clcf1, Tgm1, Ptx3, S100a10, Sphk1) and secrete protective mediators such as TGF-β, IL-10, BDNF, IGF-1, thrombospondins, and estrogen, supporting neuronal survival, synaptogenesis, and plasticity and attenuating local inflammation (Kisucká et al., 2021; Liu et al., 2024; Manzhulo et al., 2025). Structurally, A2 astrocytes can form relatively organized scars and perivascular barriers via STAT3-dependent mechanisms, limiting inflammatory infiltration and lesion spread and protecting surrounding neural tissue (Okada et al., 2006; Guo et al., 2021). A2 astrocyte-derived TGF-β can also suppress microglial M1 polarization and promote M2-like repair states, reshaping immune microenvironments through intercellular interactions (Kisucká et al., 2021).

#### 2.2.2 Astrocyte immunometabolism and changes in the inflammatory microenvironment

In AD, a disease marked by chronic inflammation and metabolic dysfunction, astrocytes progressively evolve from classical “metabolic support cells” into integrative hubs that couple energy metabolism with immune signaling. Under physiological conditions, astrocytes regulate glucose/lactate metabolism, lipid and cholesterol synthesis, glutamate–glutamine cycling, and antioxidant systems to provide neuronal substrates and maintain synaptic and perivascular microenvironmental homeostasis (Beard et al., 2020; Huang et al., 2021). Yet, under persistent stimulation by Aβ deposition, tau aggregation, and peripheral inflammatory signals, astrocytes undergo profound metabolic reprogramming. Dysregulated carbohydrate and lipid metabolism, mitochondrial dysfunction, and redox imbalance become tightly coupled with inflammatory pathways, generating an immunometabolic disequilibrium that reshapes the brain’s immune milieu.

At the level of energy metabolism, astrocytes are key components of the astrocyte-neuron lactate shuttle(astrocyte-neuron lactate shuttle, ANLS). Their high glycolytic flux and lactate export provide oxidative substrates to neurons and support network activity and synaptic plasticity (Rabah et al., 2023; Zhu et al., 2025). In early AD, Aβ and inflammatory signals impair astrocytic glucose uptake and downstream pathways, manifesting as reduced glycolysis and TCA efficiency, diminished lactate output, impaired oxidative phosphorylation, and excessive ROS generation (Barnett et al., 2025; Li et al., 2025). Although increased glycolysis and metabolic reprogramming may initially serve compensatory roles, chronic stimulation overwhelms NADPH-dependent antioxidant defenses and glutathione metabolism. Transcription factors such as NF-κB and STAT3 remain persistently activated, shifting astrocytes toward A1-like pro-inflammatory states and promoting production of IL-1β, IL-6, TNF-α, and complement C3, thereby converting metabolic imbalance into amplified neuroinflammation (Boonpraman and Yi, 2024; Rabah et al., 2025; Shafi et al., 2025).

Lipid metabolic dysregulation represents another major axis of astrocyte immunometabolic dysfunction in AD. Astrocytes are principal sources and distributors of cholesterol and fatty acids in the brain. They package lipids into APOE-containing lipoprotein particles for delivery to neurons and endothelial cells, while forming lipid droplets to buffer the toxicity of free fatty acids and oxidative products (Qi et al., 2021; Windham et al., 2024). In high-risk APOE4 contexts, astrocytes show defects in fatty acid uptake, β-oxidation, and lipid droplet turnover, resulting in lipid droplet accumulation, mitochondrial dynamical abnormalities, and increased lipid peroxidation. These changes weaken detoxification of neuron-derived toxic fatty acids and promote release of lipotoxic mediators (e.g., long-chain saturated fatty acids, oxidized phospholipids), directly injuring neurons and activating microglia (Sienski et al., 2021; Haney et al., 2024). Although early lipid droplet formation can transiently sequester excess lipids and limit peroxidation, when buffering capacity saturates—or when APOE4 impairs lipid efflux and redistribution—astrocytes become sources of lipid-derived DAMPs. Through activation of Toll-like receptors, complement receptors, and inflammasome-related signaling (e.g., NLRP3), metabolic stress is converted into sustained inflammatory output (Haney et al., 2024).

Within the immune microenvironment, immunometabolically reprogrammed astrocytes act as major producers of cytokines and chemokines. Transcriptomic studies indicate that A1-like astrocytes in AD upregulate complement C3 and related cascade genes. Astrocyte-derived C3 can act on C3aR in microglia and neurons, driving synaptic pruning and spine loss, sustaining pro-inflammatory microglial activation, and accelerating cognitive decline (Lian et al., 2015; Lian et al., 2016). In parallel, inflammatory and chemotactic signals from reactive astrocytes can weaken BBB integrity and promote peripheral immune cell infiltration, transforming Aβ-associated focal inflammation into sustained chronic immune responses (Sweeney et al., 2018).

Importantly, immunometabolic imbalance is not confined to astrocytes alone. Through crosstalk with microglia, neurons, and peripheral immune cells, astrocytes shape a highly dynamic and spatially heterogeneous immune microenvironment. Microglial release of C1q, TNF-α, and IL-1α induces astrocytes toward A1-like neurotoxic states (Liddelow et al., 2017); conversely, astrocyte-derived complement and lipid signals can maintain microglial pro-inflammatory activation, forming self-amplifying inflammatory loops (Lian et al., 2015; Guttenplan et al., 2020). Chronic immunometabolic stress can also drive subsets of astrocytes into senescence, with p16INK4a and γH2AX upregulation and accumulation of lipid peroxidation products such as 4-HNE and MDA. Senescent astrocytes can sustain inflammatory output via senescence-associated secretory phenotypes (SASP), releasing IL-6 and IL-8 and worsening local inflammatory–metabolic environments, generating hard-to-reverse “inflammatory memory” (Bhat et al., 2012; Bussian et al., 2018). Thus, astrocyte immunometabolic remodeling in AD not only compromises metabolic support and neuroprotection but also reprograms immune microenvironments to promote disease initiation and progression.

## 3 Exercise modalities in AD: reported benefits and differences in the level of evidence

Although several exercise modalities appear beneficial in AD, the level of evidence is not uniform. Astrocyte-focused mechanistic evidence is strongest for aerobic exercise and HIIT in rodent models, whereas evidence for resistance and mind-body exercise is derived primarily from clinical outcomes or indirect biomarkers rather than cell-type-specific readouts.

### 3.1 Aerobic exercise

Aerobic exercise is the best-characterized modality in AD models and currently provides the most direct evidence for exercise-related changes in astrocyte metabolism, AQP4 localization, and neuroinflammatory signaling, although most of these data come from rodents. Mechanistically, regular aerobic training increases cerebral blood flow, enlarges hippocampal volume, boosts mitochondrial count, and promotes neurogenesis, thereby enhancing synaptic plasticity (Pahlavani, 2023; Kaagman et al., 2024). Clinical studies suggest that long-term aerobic exercise may improve some cognitive, functional, or quality-of-life outcomes, whereas the strongest astrocyte-specific mechanistic evidence currently comes from rodent paradigms such as treadmill running and swimming (Yang et al., 2022).In animal models of AD, sustained aerobic training reduced Aβ pathology: aerobic exercise enhanced glymphatic clearance of Aβ in the hippocampus, improving learning and memory (Liang et al., 2025). Aerobic interventions also modulate peripheral biomarkers; exercise increased neuron-derived extracellular vesicles in blood, elevating circulating brain-derived neurotrophic factor (BDNF) and Humanin levels associated with neuroprotection (Delgado-Peraza et al., 2023). Additionally, aerobic training can ameliorate mitochondrial dysfunction in AD models by facilitating the transfer of healthy mitochondria from astrocytes to neurons, promoting neuronal repair (Cai et al., 2025). Aerobic exercise may also exert systemic effects; in early-stage AD mice, it reshaped the gut microbiota profile (favoring lipid and bile acid metabolism pathways) correlated with slowed cognitive decline (Wei et al., 2025). Collectively, these findings underscore that regular aerobic exercise provides multidimensional benefits—improving neurological function, enhancing neuroplasticity, and potentially modifying disease risk factors in neurodegenerative conditions.

### 3.2 High-intensity interval training

High-intensity interval training (HIIT), which alternates short bouts of vigorous exercise with recovery periods, is emerging as a time-efficient intervention with promising mechanistic effects in animal models, including altered astrocyte phenotype and AQP4 polarization; however, clinical evidence in AD remains sparse. HIIT can induce neurophysiological responses equal or superior to continuous moderate exercise in a shorter duration (Schenkman et al., 2018). Some evidence even suggests HIIT confers greater benefits than steady moderate aerobic exercise for certain brain outcomes. In animal research, HIIT stimulated more robust adult neurogenesis in the hippocampus than medium-intensity training (Lambertus et al., 2024). Mechanistically, HIIT’s repetitive vigorous muscle contractions release a surge of myokines and exercise factors that cross the blood–brain barrier. For example, HIIT elevates peripheral lactate and irisin, which can reduce Aβ accumulation and promote neurogenesis, overlapping with pathways seen in aerobic training but achieved more efficiently (Van Wegen et al., 2020). In an AD rat model, eight weeks of HIIT improved behavioral outcomes and, when combined with the compound ecdysterone, synergistically ameliorated hippocampal oxidative stress and prevented neuron loss (Gholipour et al., 2022). Other mouse studies show region-specific metabolic effects of HIIT: HIIT modulated astrocyte–neuron energy metabolism in the hippocampus and hypothalamus of APP/PS1 AD mice, corresponding with alleviated behavioral deficits (Ying et al., 2023). Notably, HIIT appears to influence the brain’s waste clearance systems. One study found HIIT training altered astrocyte phenotype and polarization of aquaporin-4 channels, enhancements that facilitated the glymphatic and renal clearance of Aβ and hyperphosphorylated tau in an AD model (Feng et al., 2023). Long-term HIIT has also been linked to upregulating angiogenic and neuroplastic pathways; aged mice undergoing HIIT exhibited activation of brain vascular growth signals and synaptic plasticity, alongside improvements in cognitive performance (Lei et al., 2024). Even blood plasma harvested from HIIT-trained animals conveys benefits: transfusion of “exercised” plasma increased hippocampal neurogenesis and improved memory in AD model rats (Norevik et al., 2024). Overall, HIIT is a promising and time-efficient modality, but current support for astrocyte-related mechanisms in AD remains largely preclinical and is not yet sufficient to establish modality-specific clinical recommendations.

### 3.3 Resistance exercise

Resistance training (RT) is increasingly recognized for cognitive, vascular, and anti-inflammatory benefits in aging, but direct evidence linking RT to astrocyte remodeling in AD remains limited. Unlike aerobic exercise, resistance training engages the muscle–brain axis more directly: muscle contractions during RT stimulate the release of myokines and growth factors that influence central nervous system function and plasticity (Cámara-Calmaestra et al., 2025). At a molecular level, moderate- to high-intensity RT elevates levels of BDNF in both blood plasma and the brain, supporting hippocampal and cortical plasticity (Liu et al., 2022a). Resistance exercise also exerts anti-inflammatory effects by lowering pro-inflammatory cytokines (like TNF-α, IL-6) and increasing anti-inflammatory cytokines (such as IL-10), thereby mitigating chronic neuroinflammation that contributes to neurodegeneration (Marston et al., 2017). Animal research reinforces these benefits: in rodent models, resistance exercise reduced neuropathology by promoting Aβ clearance, decreasing amyloid plaque burden, and attenuating hyperphosphorylated tau accumulation (Setayesh and Mohammad Rahimi, 2023). Notably, preconditioning with resistance exercise has been shown to protect the aging brain under stress. In aged mice, a short “prehabilitation” RT regimen before surgery prevented postoperative cognitive decline by reducing neuroinflammatory markers and improving mitochondrial health via activation of the PGC-1α/BDNF pathway (Liu et al., 2022a). Similarly, resistance exercise in naturally aged rats upregulated Notch signaling to enhance neuronal autophagy, which correlated with better cognitive performance (Chen et al., 2025). Cerebrovascular improvements also accompany RT: a 12-week structured RT program increased resting blood flow in memory-related regions (e.g. hippocampus, posterior cingulate) and improved cerebral vascular reactivity in older adults (Macaulay et al., 2022).

### 3.4 Mind-body exercise

Mind-body exercises such as yoga, tai chi, and qigong may improve cognition, mood, balance, and sleep in older adults, yet astrocyte-specific mechanisms in AD remain largely inferential. These gentle practices are particularly well-suited for older individuals and neurodegenerative disease patients due to their low impact and holistic benefits. Although challenging to replicate in animal models, clinical research highlights significant improvements in both neurological and psychological outcomes from mind-body interventions (Zhang et al., 2024). A key advantage of mind-body exercise is its regulation of the autonomic nervous system and stress response. Regular practice can reduce sympathetic overactivity, lower cortisol levels, and improve mood and sleep quality, thereby addressing non-motor symptoms like anxiety and depression that commonly co-occur with conditions such as AD and PD (Nguyen et al., 2024). In Alzheimer’s and dementia care, gentle dyadic tai chi (performed with caregiver participation) has been associated with better functional mobility and balance. Participants with dementia who engaged in a 16-week tai chi program showed improved single-leg stance time and Timed-Up-and-Go scores, indicating enhanced stability and decreased fall propensity (Yao et al., 2013). Yoga interventions likewise show promise in those at risk for or living with neurodegenerative conditions. In a study of older women at risk for AD, a 12-week Kundalini yoga program led to significant improvements in subjective memory complaints and executive functioning, comparable or superior to a memory-training control group (Grzenda et al., 2024). Furthermore, yoga produced unique immunological signatures: practitioners had altered expression of inflammation-related genes and lower levels of age-related chemokines relative to controls, suggesting an anti-inflammatory effect underlying the cognitive benefits (Grzenda et al., 2024). Neuroimaging evidence supports yoga’s neuroprotective role: a randomized trial using MRI found that yoga practice over 6 months prevented gray matter atrophy in women at high AD risk, in contrast to control subjects who showed decline in hippocampal and frontal gray matter volume (Krause-Sorio et al., 2022). Complementary findings show that long-term tai chi practice can induce structural brain changes; older adults training in tai chi exhibited strengthened white matter connectivity in brain networks subserving memory and motor control (Yue et al., 2020). In sum, mind-body exercises offer multidimensional therapeutic effects – they not only target motor and cognitive impairments in neurodegenerative diseases, but also alleviate stress, improve cardiopulmonary function, and enhance brain structure and function. By synchronizing movement with breath and attention, these practices provide a gentle, integrative approach that addresses both the neural and mental health aspects of neurodegenerative disorders.

Taken together, current evidence supports tailored, multimodal exercise programs, but it would be premature to assume that all exercise modalities exert equivalent astrocyte-mediated effects in AD.

## 4 Exercise may influence astrocyte-associated pathways relevant to AD

Exercise is increasingly investigated as an adjunctive intervention in AD, and current evidence—predominantly from animal models—suggests that its neuroprotective effects may involve astrocyte-associated remodeling rather than purely neuron-autonomous mechanisms. These astrocyte-associated responses can be organized into several overlapping domains, including regulation of reactive inflammatory programs, metabolic support, AQP4-associated perivascular clearance, and astrocyte–microglia crosstalk.

### 4.1 Exercise regulates astrocyte polarization

Disturbances in reactive astrocyte programs are a core feature of AD-related neuroinflammation, but these programs should be viewed as a continuum rather than a strict A1/A2 dichotomy. Under Aβ stimulation, astrocytes often shift toward neurotoxic A1-like states, while A2-like protective functions are compromised. Evidence from preclinical models indicates that moderate-intensity aerobic exercise can reduce the expression of A1-like reactive markers and enhance protective astrocyte-associated programs, which is broadly consistent with a shift toward a less neurotoxic phenotype (Belaya et al., 2020; Jiang et al., 2021). In APP/PS1 transgenic AD mice, long-term treadmill training significantly downregulated hippocampal A1 markers (e.g., complement C3, Serping1) while upregulating A2-associated genes (e.g., S100A10, PTX3), accompanied by improved performance in cognitive behaviors (Belaya et al., 2020). Exercise-induced changes in GFAP require cautious interpretation, because increased, decreased, or normalized GFAP expression may each reflect context-dependent astrocyte remodeling rather than uniformly beneficial or harmful activation. Voluntary running also suppressed expression of C3 and S100B in prefrontal astrocytes and increased synaptic density in contact with astrocytes (Liu et al., 2023).

Exercise-induced skeletal muscle–derived factors may contribute to astrocyte remodeling. Irisin, for example, has been reported to attenuate ERK/STAT3- and NF-κB-related inflammatory signaling in astrocytes, although direct causal mediation of exercise benefits *in vivo* remains to be established (Kim et al., 2023). These signaling changes may also be associated with increased astrocyte-derived Aβ-degrading enzymes, such as neprilysin, and with AMPK-related transcriptional remodeling; however, support for these mechanisms currently comes mainly from animal and cell-based studies rather than patient data (Huang et al., 2019; Kim et al., 2023).

### 4.2 Exercise improves astrocyte–neuron metabolic coupling

A second mechanistic domain—supported mainly by preclinical studies—concerns astrocyte-mediated metabolic support to neurons, including glucose transport, lactate shuttling, and mitochondrial homeostasis. In this context, exercise may promote metabolic reprogramming that improves the efficiency and resilience of brain energy supply. Impaired brain glucose metabolism is a characteristic feature of AD (Minhas et al., 2024). Under AD pathology, severe glucose metabolic impairment compromises astrocytic energetic support, leading to abnormal lactate metabolism and neuronal injury. Appropriate exercise training can target restoration of this function (Xu et al., 2023). In preclinical models, regular exercise has been associated with increased expression of glucose transporters—especially GLUT1 and GLUT3—improved astrocytic Slc2a1-related glucose handling, and partial normalization of mitochondrial function (Pang et al., 2019; Li et al., 2025). Long-term voluntary running shows similar effects and may relate to increased TREM2 expression in microglia (Zhang et al., 2022). In diabetic rodent models with cognitive impairment, reduced hippocampal MCT2 levels suggest close coupling between cognitive deficits and glucose metabolic disorder, and exercise at sufficient intensity improves spatial memory (Shima et al., 2017). In AD pathology, reduced MCT2 expression and decreased neuronal lactate dehydrogenase A (LDHA) and B (LDHB) are closely related to astrocytic changes, implying disrupted astrocyte-mediated lactate and energy transfer; regular exercise can reverse these abnormalities (Zhang et al., 2018). Early in AD, astrocytes often transition into reactive states with increased glycolysis and lactate release (Yang et al., 2024). Moderate lactate production supports nearby neurons by providing alternative energy substrates and maintaining synaptic activity through lactate shuttling (Huang et al., 2021; Chen et al., 2025). Exercise may strengthen astrocyte–neuron lactate coupling, but whether enhanced lactate availability reflects improved metabolic support, altered signaling, or both likely depends on disease stage and exercise intensity.

### 4.3 Exercise may enhance AQP4-dependent perivascular clearance pathways

Beyond metabolic support, exercise may influence AQP4-dependent perivascular clearance pathways that contribute to solute exchange in the brain. Animal studies indicate that perivascular AQP4 localization, sleep state, vascular pulsatility, and meningeal lymphatic function jointly shape brain solute transport; accordingly, exercise-related effects on clearance are unlikely to depend on AQP4 alone (Simon et al., 2022; Li et al., 2025). In AD patients and models, AQP4 polarization is often impaired—normally AQP4 is highly enriched at perivascular astrocytic endfeet, but in AD this polarized localization is disrupted, decreasing glymphatic clearance efficiency and facilitating Aβ deposition (Liu et al., 2022b). In APP/PS1 mice, sustained exercise has been associated with reduced Aβ burden, decreased glial reactivity, and improved perivascular localization of AQP4, with loss-of-function studies supporting a contributory role for AQP4 in these benefits (Liu et al., 2022b; Feng et al., 2023) Conversely, in AQP4 knockout AD mice, exercise-induced benefits on Aβ clearance and cognitive function are abolished. HIIT may also influence astrocyte phenotypes and thereby regulate AQP4 functional localization (Feng et al., 2023). In rodent studies, these exercise-associated changes are consistent with more efficient clearance of Aβ and tau. However, there are currently no clinical studies demonstrating that exercise improves AD symptoms through modulation of the glymphatic system; nevertheless, this possibility offers a new perspective for further clinical exploration of therapeutic mechanisms and interventions in AD.

### 4.4 Exercise reshapes inflammatory microenvironments and glial crosstalk

Exercise may also reshape the inflammatory microenvironment by weakening maladaptive astrocyte–microglia feedforward loops and enhancing endogenous antioxidant programs. Preclinical studies suggest that exercise can reduce pro-inflammatory astrocyte-associated markers in the CNS (Yang and Zhuang, 2025), including GFAP, C3, IL-1β, and TNF-α, while shifting glial signaling toward a less inflammatory state in rodent AD models (Belaya et al., 2020; Green et al., 2024). In AD contexts, this may interrupt excessive complement cascade activation and reduce microglia-mediated overpruning of synapses, thereby protecting synaptic connectivity. Recent human biomarker and PET studies support the clinical relevance of Aβ–microglia–astrocyte interactions in AD, underscoring the translational importance of this axis even though exercise-specific human data remain limited (Ferrari-Souza et al., 2026).

Exercise-induced activation of nuclear factor erythroid 2–related factor 2 (Nrf2) can strengthen antioxidant and anti-inflammatory capacities in astrocytes and microglia (Kanninen et al., 2009). Exercise promotes nuclear translocation of Nrf2 and activation of antioxidant response elements (ARE), upregulating antioxidant enzymes and inhibiting excessive assembly of the NLRP3 inflammasome (Choi et al., 2025; Lee et al., 2025). Reports suggest that moderate exercise inhibits NLRP3 activation via the Nrf2-TXNIP-Trx pathway, and interventions targeting the Nrf2-TXNIP-TrX axis similarly modulate NLRP3 in glial cells (Wang et al., 2019; Weng et al., 2023). Thus, exercise improves inflammatory environments both by reducing pro-inflammatory signal sources and by engaging endogenous anti-inflammatory programs. BDNF rises after exercise and not only directly promotes synaptic plasticity and neurogenesis, but may also act on astrocytes to enhance trophic support to neurons. Multiple exercise modes increase serum and brain BDNF, improving astrocyte activation states and AD outcomes (Liu et al., 2022; Xiao et al., 2025). β-hydroxybutyrate, an important energy substrate and signaling molecule, increases during exercise and can activate astrocytic GPR109A, suppressing NF-κB signaling and reducing iNOS, COX-2, and inflammatory cytokines such as TNF-α, IL-1β, and IL-6 (Fu et al., 2015; Huang et al., 2022). IL-6 shows complex dynamics: although it transiently increases during vigorous exercise, acute elevations may trigger anti-inflammatory cascades—stimulating IL-1 receptor antagonist (IL-1ra) and IL-10 release and suppressing TNF-α production (Petersen and Pedersen, 2005; Mu et al., 2022; Plantone et al., 2025).

Nevertheless, direct evidence that exercise reprograms astrocyte-microglia interactions in humans is still limited. Future studies should combine exercise interventions with plasma or CSF biomarkers of glial activity, multimodal neuroimaging, and stratification by sex and APOE genotype to determine whether specific exercise prescriptions truly modify inflammatory crosstalk in patients with prodromal or established AD.

## 5 Discussion

Astrocytes have emerged as plausible mediators of exercise-associated neuroprotection in Alzheimer’s disease (AD), yet the current mechanistic foundation remains dominated by animal data. Rather than viewing exercise as a single intervention with uniform effects, it is more accurate to regard it as a family of stimuli that may influence astrocyte biology through partially overlapping pathways, including inflammatory phenotype regulation, metabolic support, perivascular clearance, and glial crosstalk (Belaya et al., 2020; Feng et al., 2023; Xu et al., 2023). Accordingly, exercise should be considered a promising adjunctive strategy rather than a stand-alone cure, with potential relevance to the multifactorial nature of AD pathogenesis (López-Ortiz et al., 2021; Zhao, 2024; Zhang et al., 2025).

In several preclinical models, exercise has been associated with reduced expression of A1-like reactive markers, improved astrocyte-neuron metabolic coupling, better preservation of synaptic function, and improved perivascular localization of AQP4 (Belaya et al., 2020; Rabah et al., 2025). These changes are broadly consistent with lower inflammatory tone, more efficient substrate delivery, and enhanced brain solute transport. However, these observations do not justify a uniform claim that exercise “suppresses” astrocyte activation or “restores” glymphatic function in humans. A more cautious interpretation is that exercise may remodel astrocyte-associated pathways in ways that are compatible with neuroprotection, while the magnitude, timing, and cellular specificity of these effects remain incompletely defined, especially in clinical populations (Huang et al., 2019; Liu et al., 2023).

Importantly, the A1/A2 framework used throughout this review should be interpreted as a heuristic rather than a complete biological taxonomy. Recent single-nucleus and spatial transcriptomic studies indicate that astrocytes in AD occupy multiple region- and stage-specific states with overlapping inflammatory, metabolic, synaptic, and vascular programs (Green et al., 2024; Serrano-Pozo et al., 2024; İlğun and Çakır, 2025). Therefore, future work should move beyond binary labeling and identify exercise-responsive astrocyte states using multi-omic, spatially resolved, and temporally informed approaches. Such strategies will be essential for clarifying whether exercise truly shifts astrocytes toward more protective functional states, or instead remodels distinct subsets of reactive astrocytes in a context-dependent manner (Endo et al., 2022; Green et al., 2024; Serrano-Pozo et al., 2024).

Several translational gaps also warrant emphasis. First, most astrocyte-focused exercise data arise from rodent models, whereas human astrocytes differ substantially in morphology, molecular diversity, and network integration (Ciuba et al., 2025). In clinical settings, available evidence has mainly involved cognitive outcomes, neuroimaging changes, or systemic biomarkers rather than direct astrocyte-specific readouts (López-Ortiz et al., 2021; Zhao, 2024). Second, discussion of glymphatic mechanisms should remain appropriately cautious. Although exercise improves AQP4 localization and tracer-based clearance measures in several rodent studies, the quantitative contribution and biophysical basis of glymphatic flow in the human brain remain unsettled, and clearance is likely shaped by sleep, vascular pulsatility, blood-brain barrier integrity, and meningeal lymphatic drainage rather than by AQP4 alone (Liu et al., 2022b; Simon et al., 2022). Third, the astrocyte-related effects of exercise are unlikely to be equivalent across modalities. Current studies vary substantially in modality, intensity, duration, timing, and adherence, making direct comparison difficult and limiting the precision of clinical recommendations (López-Ortiz et al., 2021; Zhao, 2024). Future studies should therefore directly compare aerobic, resistance, interval, and multimodal training paradigms using biomarker-informed designs and standardized outcome measures. Bridging preclinical findings with human trials will be essential to develop evidence-based exercise guidelines for patients with prodromal or established AD.

Key biological modifiers also deserve greater emphasis. Sex influences AD risk, tau burden, immune responses, and astrocyte-associated biomarkers, whereas APOE4 alters astrocytic fatty-acid trafficking, lipid-droplet biology, and inflammatory tone; these factors may also modify responsiveness to exercise. Future mechanistic and interventional studies should therefore stratify participants by sex and APOE genotype, or at minimum adjust for these variables analytically.

In summary, targeting astrocytes through exercise represents a promising strategy for AD prevention and therapy. As an important complement to pharmacotherapy, regular physical activity can mitigate AD pathology and improve cognition via multi-level effects on glial cells. Of course, exercise is not a cure-all, and its benefits may depend on individual variability and disease stage. Nevertheless, current evidence supports exercise as a safe, cost-effective, and practical strategy. As the field moves away from single-pathology approaches toward holistic strategies that regulate neuroinflammation and enhance neural plasticity, astrocyte-focused exercise interventions align well with this shift. Overall, exercise should be considered a promising adjunctive strategy rather than a stand-alone cure, with future progress depending on rigorous mechanistic studies, genotype- and sex-aware trial design, and clinically translatable astrocyte biomarkers.
